# Histoprotective Effect of Essential Oil from *Citrus aurantifolia* in Testosterone-Induced Benign Prostatic Hyperplasia Rat

**DOI:** 10.1155/2019/3031609

**Published:** 2019-09-25

**Authors:** Desmond O. Acheampong, Isaac K. Barffour, Alex Boye, Ernest A. Asiamah, Francis A. Armah, Christian K. Adokoh, Joy F. Oluyemi, Benjamin Adrah, Richard Opoku, Emmanuel Adakudugu

**Affiliations:** ^1^Department of Biomedical Sciences, School of Allied Health Sciences, University of Cape Coast, Cape Coast, Ghana; ^2^Department of Medical Laboratory Science, School of Allied Health Sciences, University of Cape Coast, Cape Coast, Ghana

## Abstract

**Background:**

Benign prostatic hyperplasia (BPH) is a common urological disorder reported among ageing men.

**Objective:**

The study assessed histoprotective effect of lime essential oil (LEO) in a rat model of testosterone-induced benign prostatic hyperplasia (BPH) and evaluated its ability to reverse testosterone-mediated changes in the testis, kidney, and liver.

**Materials and Methods:**

Adult Sprague Dawley (aged 12 weeks, 240–390 g) male rats were intramuscularly injected with testosterone enanthate (TE) (10 mg/kg) reconstituted in olive oil for ten days to establish benign prostatic hyperplasia (serum PSA level ≥ 1.24 ng/ml) in. After confirmation of BPH (sustained serum PSA level ≥ 1.24 ng/ml), rats in all groups (LEO: 30, 100, and 300 mg/kg, *po*, *n* = 6; finasteride: 15 mg/kg, *po*, *n* = 6) except model (BPH without treatment) and sham (no BPH and no treatment) groups were treated for 21 days. At the end of treatment, rats were anesthetised and blood was collected via cardiac puncture to determine serum PSA and total antioxidant capacity (TAC) levels. The prostate gland, testis, kidney, and liver were harvested, weighed, histologically processed and stained with H&E.

**Results:**

LEO- and finasteride-treated groups recorded lesser mean prostatic weights relative to their model group. Baseline mean serum PSA level of LEO- and finasteride-treated groups reduced significantly (*p* < 0.05) relative to model group. Serum TAC levels were also higher in LEO- and finasteride-treated groups relative to model group. LEO-treated groups had less thickened glandular epithelium, smaller acini, fewer prostatic secretions and more fibromuscular stroma relative to model group. LEO and finasteride treatment produced improved histomorphological characteristics of testis, kidney, and liver compared to model group.

**Conclusion:**

By the current results, *Citrus aurantifolia* LEO may possess active agents that can be explored for translational medicine against BPH.

## 1. Introduction

Benign prostatic hyperplasia (BPH) is a common urological disorder reported among ageing men [1]. The disorder is a non-malignant uncontrolled proliferation of the parenchymal cells of the prostate gland, resulting in the enlargement of the prostate gland [2]. The prostate gland is a critical reproductive organ in males and consists of branched tubuloacinar glandular epithelium embedded in a fibromuscular stroma. Secretion from the gland contributes one-fifth to one-third (20–33.3%) of seminal volume, and it is necessary for sperm activation owing to the composition of its secretions including citrate and enzymes such as fibrinolysin, hyaluronidase, and acid phosphate [3]. The pathophysiology of BPH is not clearly understood; however, its etiology is attributable to inflammatory [4] and/or oxidative stress [5]. Inflammation is known to have an association with excessive cell proliferation and death as compensatory hyperplasia could occur after cell death from chronic inflammation [6]. Oxidative stress also generates unstable chemical species that cause damage to mRNA, DNA, and proteins involved in cell death and proliferation [7]. Thus, antioxidant-rich and/or anti-inflammatory drug especially those of natural product origin may prevent or delay the progression of BPH. Interestingly, essential oils from *Citrus aurantifolia* have demonstrated both anti-inflammatory and antioxidant properties.

Lime essential oil is obtained from fruit peels of the *Citrus aurantifolia*. Lime essential oils have been investigated for their medicinal properties [8–11]. Nonetheless, its effect on uncontrolled BPH is yet to be studied and explored. Lime essential oil is locally used as a sweetener in the food industry but has recently been shown to possess antimicrobial [12, 13], anti-inflammatory [13], anticancer [8] and antioxidative [14] properties attributable to its phytochemical constituents. Thus, lime essential oil may be able to ameliorate variables which are indicative of benign prostatic hyperplasia. The study investigated the protective potential of lime essential oil (LEO) on the prostate gland in Sprague Dawley male albino rats experimentally induced with BPH.

BPH is also characterized by high serum and plasma testosterone levels. Hypertestosteronemia is reported to cause damage in the kidney, testis, and possibly, other body organs [15, 16]. The study also aimed at investigating if lime essential oil could ameliorate testosterone-induced histological changes in selected organs including the prostate gland, testis, liver, and kidney.

## 2. Materials and Methods

### 2.1. Drugs and Chemicals

Testosterone was purchased from ADD Pharma (GH) LTD, Accra, Ghana. Finasteride was purchased from Cape Coast Teaching Hospital pharmacy, Abura, Cape Coast, Ghana. Chemicals used in the study included phosphomolybdenum, a reagent prepared by mixing in 1 : 1 : 1 ratio, ammonium molybdate (4 mM), sodium phosphate (28 mM), and sulphuric acid (0.6 M), and ascorbic acid (20–200 *μ*g/mL) from the biochemistry department, University of Cape Coast, Ghana.

### 2.2. Preparation of Essential Oil from *Citrus aurantifolia* Fruits

Oil was extracted from the unripened fruit peels of *Citrus aurantifolia* by hydrodistillation using a modified Clevenger apparatus. The unripe fruits of *Citrus aurantifolia* of the family Rutaceae were collected from Amissakrom Ekroful in the Mfantseman West District of the Central Region, Ghana. The samples were examined and authenticated at the Herbarium Unit of School of Biological Sciences, University of Cape Coast, Ghana, where a voucher specimen (SBS/UCC/102) was deposited.

### 2.3. GC/Mass Spectrometry Analysis of the Unripe LEO Chemical Composition

The LEO was stored in a refrigerator at 4°C prior to Gas chromatography Mass Spectrometry (GC-MS) analyses using a Hewlett Packard 6890 Series, equipped with a HP Chemstation data processor, fitted with a HP-Innowax bonded phase capillary column: 30 m × 320 *μ*m id. and 0.50 *μ*m film thickness (Hewlett Packard, Palo Alto, USA). The Column temperature was 40°C (8 min.) to 180°C at 3°C/min., 180–230°C at 20°C/min., 230°C (20 min.); injector temperature was 250°C and a detector temperature of 250°C was used. Carrier gas was H_2_ (34 kPa), and split ratio 1 : 50, volume injected was 1 *μ*L and was diluted in hexane (1 : 10) for hydrodistillation samples and 1 *μ*L neat for supercritical extraction samples.

The GC/MS analysis was done in a 6890/MSD5973 Hewlett gas chromatograph with a mass selective detector, equipped with HP Chemstation software and using Wiley 275 spectra data [17] with few modifications. Briefly, a fused silica capillary column HP-Innowax: 30 m × 250 *μ*m, 0.25 *μ*m film thickness (Hewlett Packard, Palo Alto, USA) was used. The programmed temperature for GC/MS was the same as that of the GC analysis. The interface temperature and split ratio were 280°C and 1 : 100 respectively. The carrier gas He (56 kPa), flow rate: 1.0 mL/min., ionization energy 70 eV, mass range 40–350, volume injected 0.5 *μ*L, and solvent cut 3.5 min. were used. The characterization of individual components was based on comparison of their GC retention indices (RI) on polar columns and comparison of their mass spectra by GC/MS to those described in literature.

### 2.4. Induction of BPH in Rats

Thirty-six (36) healthy adult male Sprague Dawley rats aged 12-week-old and weighing 240–390 g were purchased from the Animal Breeding Unit of Noguchi Memorial Institute for Medical Research, University of Ghana, Legon, Ghana, and transported to the Animal House of the School of Biological Science, University of Cape Coast, Ghana, where the experiment was conducted. The rats were allowed two weeks to acclimatize to laboratory conditions before commencement of the experiments under ambient conditions of temperature, relative humidity, and 12 hours light/dark cycle. They were provided with a standard pellet diet (Grower Mash, Essaar, Ghana) and water *ad libitum*.

BPH was established in 30 adult male rats by injecting (IM) testosterone enanthate (10 mg/kg) reconstituted in olive oil in alternate thighs of each rat for ten days. The remaining six rats were injected (IM) with olive oil (5 ml/kg) on alternate thighs for ten days. On the 11^th^ day, rats were anesthetised with petroleum ether, and a cardiac puncture was carried out by a trained technician to collect blood for baseline serum PSA level determination. All the rats injected with testosterone enanthate had a serum PSA ≥ 1.24 ng/ml and were randomly reassigned into five groups of six. Rats were treated with LEO (30, 100, and 300 mg/kg, *po*) and finasteride (15 mg/kg, *po*) daily for 21 days, while rats in the model group had no any treatment except access to food and water. Rats which received only olive oil injection had a serum PSA of ≤ 0.0010 ng/ml and were used as the sham group.

  Group 1: testosterone-enanthate + no lime essential oil/finasteride (model) (*n* = 6).
  Group 2: testosterone-enanthate + 30 mg/kg of lime essential oil (*n* = 6).
  Group 3: testosterone-enanthate + 100 mg/kg of lime essential oil (*n* = 6).
  Group 4: testosterone-enanthate + 300 mg/kg of lime essential oil (*n* = 6).
  Group 5: testosterone-enanthate + 15 mg/kg of finasteride (*n* = 6).
  Group 6: olive oil + no lime essential oil/finasteride (sham) (*n* = 6).

### 2.5. Anesthesia Induction and Isolation of Organs

At the end of 21-day treatment, all the rats were anesthetised using chloroform inhalation, and cardiac puncture was performed following thoracotomy to collect blood for serum PSA levels and total antioxidant capacity (TAC) determination. The rats were sacrificed, and some organs (prostate gland, liver, kidney, and testis) were carefully harvested for routine tissue processing and HE staining.

### 2.6. Serum PSA Measurements Using ELISA

Commercial quantitative enzyme-linked immunosorbent assay was used to determine serum PSA levels in BPH induced rats. Twenty-five microlitres (25 *μ*L) of either serum sample or calibrators (0, 2.5, 5.0, 10, 25, and 50 ng/ml of serum matrix PSA) were placed in microtitre wells precoated with 100 *μ*L of antibody-enzyme conjugate (HRP-labeled anti-mouse PSA), mixed to form the sandwich complex on the surface of the wells, and were incubated for 30 minutes at 20–25°C. Excess conjugate and unbound antigen were washed five times using 300 *μ*L solution consisting of 1 : 19 dilution of Tris-Buffered saline. A 100 *μ*L substrate-reagent mixture made up of 1.2 mM 3,3′, 5,5′- tetramethylbenzidine (TMB) and ≤6.0 mM hydrogen peroxide was then added to each well after which the total mixture was incubated at 20–25°C at 15 minutes. The reaction was halted using 100 *μ*L of 0.5 M sulphuric acid. The absorbance of the mixture was measured using Urit 680 microplate analyser at a wavelength of 450 nm. Serum PSA concentration subsequently computed from a straight line graph plotted using absorbance (as ordinate) and respective calibrator concentration (as abscissa).

### 2.7. Plasma Total Antioxidant Capacity (TAC)

Total antioxidant capacity of the plasma was determined using the phosphomolybdenum method as described by Prieto et al. [18]. A reagent solution was prepared by mixing in 1 : 1 : 1 ratio, ammonium molybdate (4 mM), sodium phosphate (28 mM) and sulphuric acid (0.6 M). Five hundred microlitres (500 *μ*L) of the plasma was mixed with 3 mL of the reagent solution in separate test tubes, and the reaction mixture was subsequently incubated at 95°C for 70 minutes. The absorbance of the mixture was measured at 695 nm using a spectrophotometer against blank after cooling to room temperature. The method was repeated using various concentrations (20–200 *μ*g/mL) of the standard, ascorbic acid. Each concentration was prepared in duplicates. A mixture containing 0.5 mL methanol and 3 mL reagent solution was used as a blank. The total antioxidant capacity (TAC) of the plasma was expressed in ascorbic acid (AscAE) equivalents.

### 2.8. Statistical Analysis

Data were analysed using GraphPad Prism 7.00 software (GraphPad, La Jolla, CA, USA). The data were expressed as means ± standard error of mean (SEM) of each group. Statistical significance was then determined using analysis of variance (ANOVA). Tests that showed a significant difference among groups were analysed by a multiple comparison procedure using Bonferroni's multiple comparison tests. The level of significance was set at *p* < 0.05.

### 2.9. Ethical Approval

Experimental animals were handled in strict compliance with the guide for the Care and Humane Use of Laboratory Animals (National Research Council, 1996) and the U. Animals (Scientific Procedures) Act, 1986, and associated guidelines, EU Directive 2010/63/EU for animal experiments.

## 3. Results

### 3.1. Chemical Composition and Characterization of LEO

The GC/MS analyses of LEO led to the identification of ten (10) phyto-constituents (Table 1). The two major isomers of germacrene, A and B, were identified (61.2%), and were followed by pinene (14%) and bornane (11%), respectively. As presented in Table 1, the remaining components were linalool dimmer (2.9%), citral (2.9%), anethole (1.5%), anisole (1.1%), 2,6-dimethylheptan-2-ol (demitol) (0.6%), and safrole (0.3%). From the GC spectrum and extracted *m*/*z* data (Table 1), the component labeled 8 with *m*/*z* 152 was identified as citral with a typical fragmentation pattern depicted in the MS spectra comparable to literature data [19]. To confirm the compounds, mechanisms of fragmentation were also computed which matched with dictionary of Natural Products online database theoretical values (http://dnp.chemnetbase.com/). According to the MS fragmentation patterns, several terpenes derivatives were identified (Table 1). Accordingly, molecules 2, 3, and 4 (supplementary data [Supplementary-material supplementary-material-1] and [Supplementary-material supplementary-material-1]) were identified as isomers of germacrene and dimmer of linalool, respectively. Presented in Table 1 is molecule 1 at retention time (Rt) 5.05 min. produced a precursor ion at *m*/*z* 136 [M* *+* *H] (C_10_H_16_). Mass spectrometer fragmentation of this molecule (supplementary data [Supplementary-material supplementary-material-1] and [Supplementary-material supplementary-material-1]) generated product ions at *m*/*z* 121, resulting from the loss of methyl (−15 Da) after a possible 1, 2 methyl rearrangement of dimethyl derivative of pinene to a more stable derivative of *β*-pinene ([Supplementary-material supplementary-material-1]). Product ions at *m*/*z* 93 also correspond to the loss of propenyl group (forty-three mass units (−43 Da)) with a bass peak of 100% intensity. The molecule was recorded as *β*-pinene since the abundance of ion *m*/*z* = 41 is more than half of the base peak whilst that for the *α*-isomer is always less than one-quarter [20]. This molecule (*m*/*z* 136) is also distinguished from other isomers such as limonene with base peak at *m*/*z* 68 [21]. Based on the individual molecular fragmentation patterns, molecules 2–10 were positively identified and characterized similarly as presented in the supplementary data (Figure and [Supplementary-material supplementary-material-1]–[Supplementary-material supplementary-material-1]). Other constituents such as demitol (*m*/*z* 145), safrole (*m*/*z* 161), anethole (*m*/*z* 147), and anisole (*m*/*z* 110) were also identified with unique fragmentation patterns as reported in Table 1.

### 3.2. Effect of LEO on the Prostate Gland

**Figure 1:**
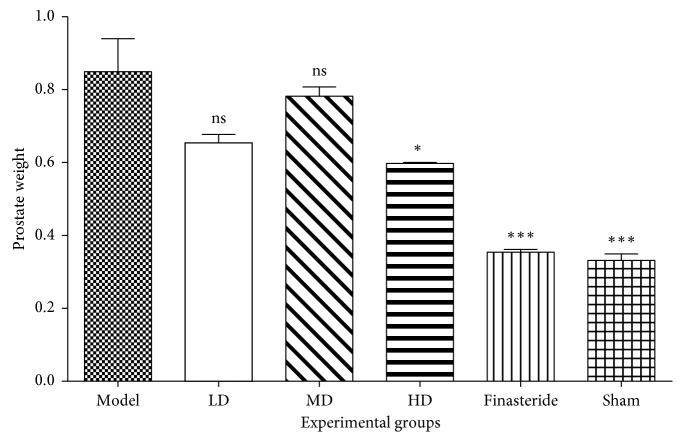
Mean prostate gland weights of experimental groups after 21 days of treatment. Data are presented as mean ± standard error of mean (*n* = 6) (ns = no significance, ^*∗*^*p* < 0.05 and ^*∗∗∗*^*p* < 0.0001). Compared to the disease control group (one way ANOVA followed by Bonferroni's post hoc). This figure demonstrates the change in prostate weights of various groups after treatment. When prostate weights were compared to disease (testosterone groups), there was a statistically significant decrease in prostate weights. Groups treated with 30 mg/kg·b.wt. and 100 mg/kg·b.wt. resulted in no significant reduction in prostate weight, while those treated with 300 mg/kg·b.wt. produced a significant decrease in prostate weight.

The mean prostate gland weights for the experimental groups (Figure 1): normal, model, low dose, middle dose, high dose, and finasteride were 0.333 ± 0.018 g; 0.853 ± 0.091 g; 0.657 ± 0.023 g; 0.785 ± 0.026 g; 0.600 ± 0.003 g and 0.356 ± 0.008 g respectively (Supplementary [Supplementary-material supplementary-material-1]). The mean prostate gland weight of testosterone-injected groups: model, LD, MD, HD, and finasteride were 155.93% (*p*=0.0001), 97.12% (*p*=0.0246), 135.52% (*p*=0.0008), 80.02% (*p*=0.0803) and 6.66% (*p*=0.9998) greater than that of normal control group respectively (Supplementary [Supplementary-material supplementary-material-1]). The mean prostate gland weights of the essential oil-treated rats were lesser than that of the model group, but the differences were not significant (*p* > 0.05). Finasteride-treated group had significantly lower mean prostate gland weight than all essential oil-treated groups (*p* < 0.05) except for the high-dose group (*p*=0.1243) (Supplementary [Supplementary-material supplementary-material-1]).

### 3.3. Effect of Treatments on Serum PSA Levels

**Figure 2:**
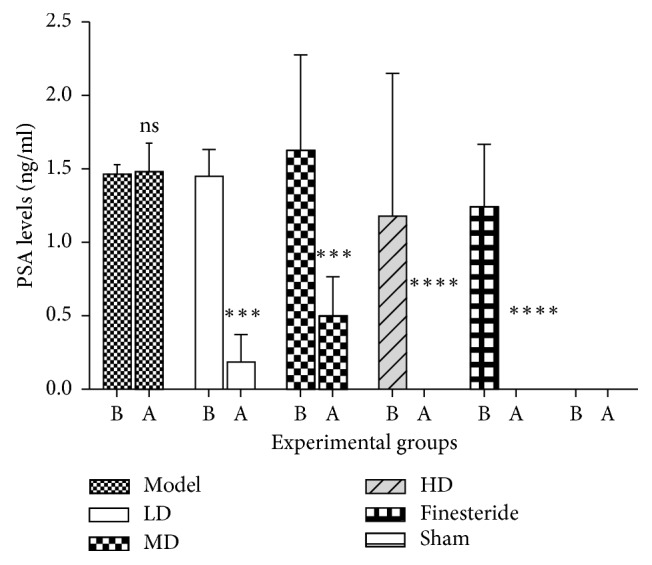
Mean serum PSA level before and after treatment for various experimental groups. Data are presented as mean ± standard error of mean (*n* = 6) (ns = no significance, ^*∗∗∗*^*p* < 0.05 and ^*∗∗∗∗*^*p* < 0.0001). B represents before treatment, and A represents after treatment. There was a decrease in the serum PSA levels of all treatment groups compared to the disease control (testosterone) group. The low-dose, mid-dose, and high-dose *Citrus aurantifolia* groups all produced a significant reduction in serum PSA levels, together with the finasteride and normal control groups. This implies that the reduction in serum PSA levels was statistically significant, upon treatment with all doses of *Citrus aurantifolia*. A greater and more significant decrease was observed in the high-dose group than the low-dose and the mid-dose groups.

The mean serum PSA levels (ng/ml) (Figure 2) of experimental groups before and after treatment were sham group (0.0010 ± 0.0002 and 0.0010 ± 0.0000), model group (1.4720 ± 0.0370 and 1.4890 ± 0.0870), LD (1.4570 ± 0.1060 and 0.1870 ± 0.1080), MD (1.6350 ± 0.2920 and 0.5020 ± 0.1550), HD (1.3450 ± 0.3960 and 0.0010 ± 0.0000), and finasteride (1.2490 ± 0.2130 and 0.0010 ± 0.0001). The mean serum PSA levels of the testosterone-injected groups were significantly greater than the normal which did not receive testosterone-injection. After twenty-one days of treatment, all the essential oil and finasteride treatment groups had their serum PSA levels significantly reduced (*p* < 0.05) except the model group. The normal group which received no testosterone injection had no significant change in the serum PSA levels.

### 3.4. Effect of Treatments on Prostate Gland Histology

**Figure 3:**
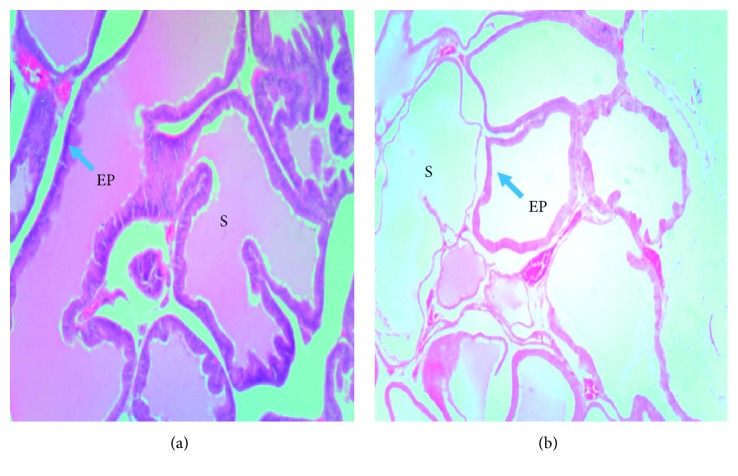
Photomicrograph of prostate glands of experimental rats after testosterone-induced benign prostatic hyperplasia: prostatic secretions (S); epithelium (EP). (a) Testosterone-injected group. (b) Sham group. The testosterone-injected group had thicker epithelium, larger acini, and more prostatic secretions. Sham group had thinner epithelium and fewer prostatic secretions.

**Figure 4:**
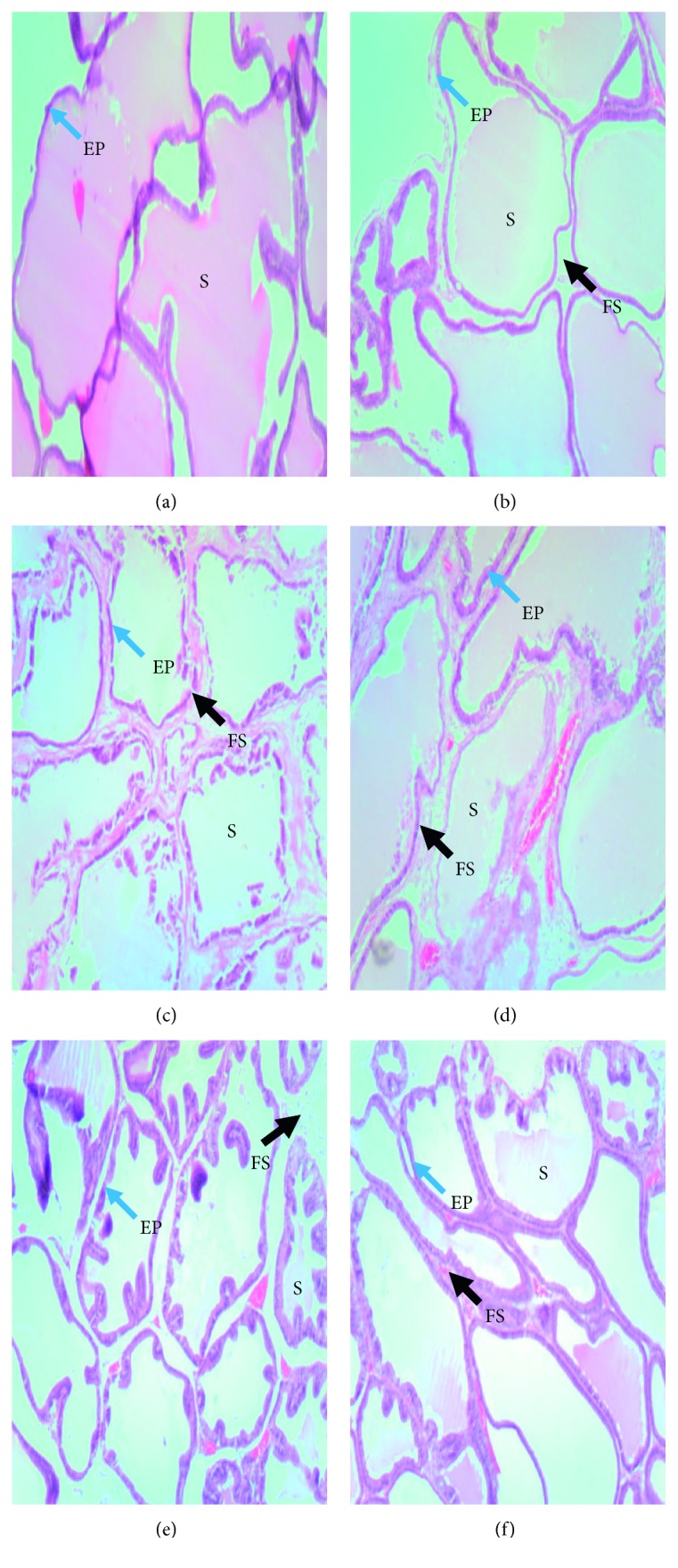
Photomicrographs of prostate glands of experimental groups after 21 days of treatment: (a) model; (b) low dose of oil (30 mg/kg); (c) mid-dose of oil (100 mg/kg); (d) high dose of oil (300 mg/kg); (e) finasteride (15 mg/kg); (f) normal control. Prostatic secretions (S), epithelium (EP), and fibromuscular stroma (FS), model group had larger acini with thicker epithelium, more prostatic secretions, and thinner fibromuscular stroma. Low dose group had slightly large acini, light prostatic secretions and thin fibromuscular stroma. Mid-dose group shows smaller acini with thinner epithelium, moderate prostatic secretion, thick strand of the fibromuscular stroma. High-dose group shows small-sized acini, moderate prostatic secretions, thin epithelium and thick fibromuscular stroma. Finasteride group had almost absent prostatic secretions and a very few amount of fibromuscular stroma, sham group had small-sized acini, little or no prostatic secretion, together with few strands of the fibromuscular stroma.

After ten days of testosterone enanthate injection, Testosterone-injected group had thicker epithelium, larger acini, and more prostatic secretions. Sham group had thinner epithelium and fewer prostatic secretions (Figure 3). After twenty-one days of treatment, testosterone-injected rats had more prostatic secretions and thinner layers of fibromuscular stroma in between acini when compared to the normal control (Figure 4). Rats which were administered with the low dose (LD), 30 mg/kg of the lime essential oil showed no significant restoration of the tissue histoarchitecture. There were very thinner strands of fibromuscular stroma in between each acinus, and more prostatic secretory activity was observed when compared to the model group. Rats in the mid-dose group (MD), 100 mg/kg, appeared to have a thick layer of fibromuscular stroma in between the acini and also had more prostatic secretions, compared to the low-dose group. Treatment with the high dose (HD), 300 mg/kg, appeared to have a thicker layer of fibromuscular stroma in between the acini, with more prostatic secretions in the acini.

### 3.5. Effect of Treatments on Total Antioxidant Capacity

**Figure 5:**
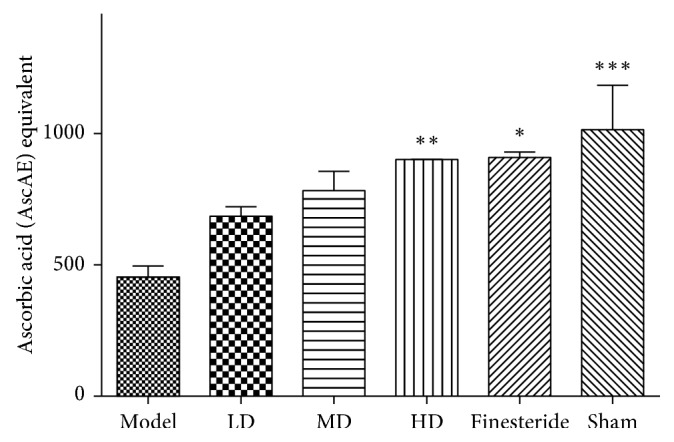
Mean plasma TAC among various treatment groups (^*∗*^*p* < 0.05 and ^*∗∗*^*p* < 0.01). Data are presented as mean ± standard error of mean. One-way ANOVA test showed a *p* value >0.05.

The mean plasma total antioxidant capacities (TAC) (AscAEq *μ*g/ml) of the experimental groups were as follows: model (453.3 ± 42.1), low dose (685.5 ± 35.61), middle dose (783.2 ± 73.14), high dose (901.6 ± 0.7782), finasteride (909.4 ± 20.44) and sham (1015 ± 168.2). The essential oil-treated groups recorded a dose-dependent increase in mean plasma TAC. One-way ANOVA test showed the mean plasma TAC of the high dose-treated, finasteride-treated and sham groups was significantly greater than the model group (*p* < 0.05) (Figure 5).

### 3.6. Effect of Treatments on Testicular Histology

**Figure 6:**
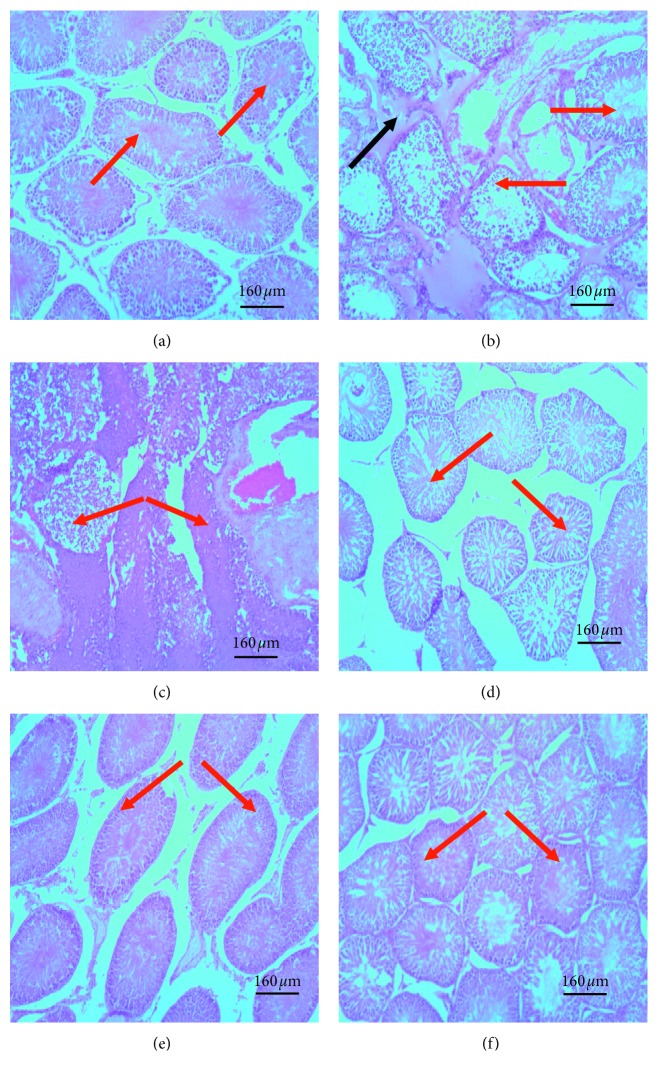
Photomicrographs of seminiferous tubules in testis of various experimental groups: (a) sham; (b) model, TE (10 mg/kg)/olive oil injection (i.m.); (c) finasteride, positive control, TE (10 mg/kg)/olive oil injection (i.m. + finasteride administration (15 mg/kg, p.o.); (d) small dose, TE (10 mg/kg) olive oil injection (i.m.) + LEO (30 mg/kg); (e) medium dose, TE (10 mg/kg)/olive oil injection (i.m.) + LEO (100 mg/kg); (f) high dose, TE (10 mg/kg)/olive oil injection (i.m.) + LEO (300 mg/kg). The red arrow represents seminiferous tubules, and blue arrow represents eosinophilic secretion in intertubular space. When compared to the sham group, model group had seminiferous tubules with less compact or eroded germinal epithelium and eosinophilic secretion in the tubulointerstitium. Finasteride-treated group had few seminiferous tubules with vacuolated epithelium. All lime essential oil-treated groups had seminiferous with intact epithelium similar to the sham group.

**Figure 7:**
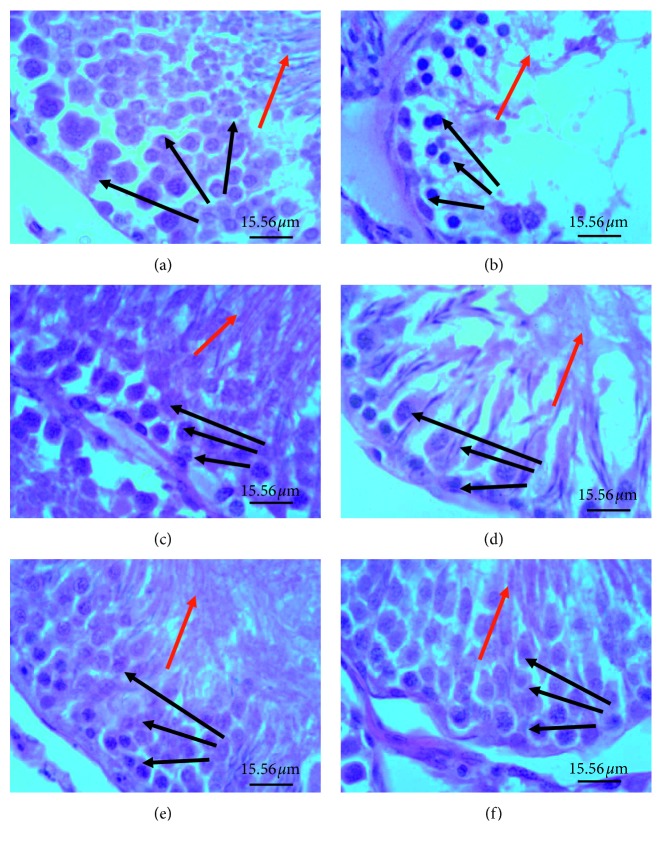
Photomicrographs of testis showing spermatogenic series in seminiferous tubules: (a) sham; (b) model, TE (10 mg/kg)/olive oil injection (i.m.); (c) finasteride (positive control), TE (10 mg/kg)/olive oil injection (i.m.) + finasteride administration (15 mg/kg, p.o.); (d) small dose, TE (10 mg/kg)/olive oil injection (i.m.) + LEO (30 mg/kg); (e) medium dose, TE (10 mg/kg)/olive oil injection (i.m.) + LEO (100 mg/kg); (f) high dose, TE (10 mg/kg)/olive oil injection (i.m.) + LEO (300 mg/kg). Red arrow represents spermatozoon in adluminal compartment, black arrows represent germinal epithelium. Model group had less compact germinal epithelium, severe pyknosis in the germinal epithelium (black arrow) and few or no spermatozoa in the adluminal compartment of the seminiferous tubules as well as eosinophilic secretion in the interstitial space. Lime essential oil treatment group had dose dependent decrease in these features.

When compared with sham group, seminiferous tubules of rats from the model group were shrunken with the germinal epithelium appearing less compact or eroded off and as an eosinophilic secretion in the intertubular space (Figure 6). Sperm cells were mostly absent in the centre of the tubules. Tubules with sperms present had an eosinophilic pithing. Also, most cells forming the germinal epithelium of their seminiferous tubules and those of the tubulointerstitium had pyknotic nuclei. The finasteride-treated group had only a few seminiferous tubules with eroded germinal epithelium. The germinal epithelia were mostly compact. Pyknotic changes in the germinal epithelium were confined to cell layer closest to the basement membrane of the seminiferous tubules. Lime essential oil-treated groups had less shrunken seminiferous tubules with compact germinal epithelium in a dose-dependent manner. Traces of pyknosis in the germinal epithelium and tubulointerstitial space were present in the medium and low dose groups but were almost absent in the high dose group (Figure 7).

### 3.7. Effect of Treatments on Liver Histology

**Figure 8:**
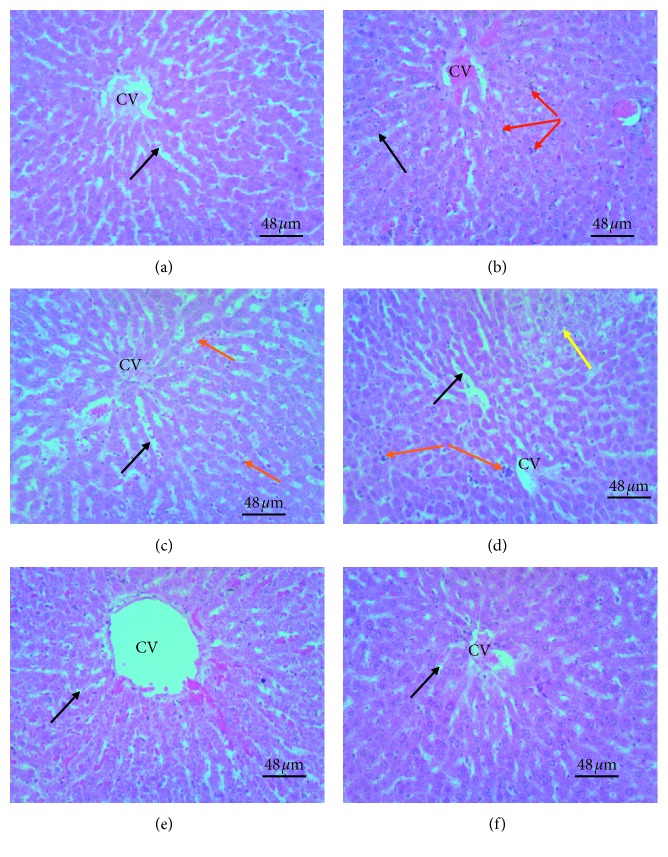
Photomicrographs of H&E stained liver sections of experimental groups: (a) sham; (b) model, TE (10 mg/kg)/olive oil injection (i.m.); (c) finasteride, positive control, TE (10 mg/kg)/olive oil injection (i.m.) + finasteride administration (15 mg/kg, p.o.); (d) small dose, TE (10 mg/kg)/olive oil injection (i.m.) + LEO (30 mg/kg); (e) medium dose, TE (10 mg/kg)/olive oil injection (i.m.) + LEO (100 mg/kg); (f) high dose, TE (10 mg/kg)/olive oil injection (i.m.) + LEO (300 mg/kg). Black arrow represents sinusoids separating plates of hepatocytes; red arrow represents Kupffer cells; Yellow arrow represents hepatocytes with fat accumulation, the orange arrow represents inflammatory cells, and CV represents centrilobular vein. The model group showed enlarged hepatocytes with eosinophilic or pale staining cytoplasm or enlarged nucleus and enlarged sinusoids infiltrated with Kupffer cells and inflammatory cells. Finasteride-treated group had dilated sinusoids with few inflammatory cells and normal sized hepatocytes with slightly enlarged nuclei with a prominent nucleolus. Lime essential oil-treated groups showed dose-dependent decrease in sinusoids infiltrated with Kupffer cells or inflammatory cells, dose-dependent increase in hepatocyte fat accumulation. High dose treated group had a presentation close to the sham group.

The model group showed enlarged hepatocytes with cytoplasmic vacuolations, an enlarged nucleus with the nucleolus and or pyknotic nuclei. Some hepatocytes appeared small and had eosinophilic cytoplasm. Sinusoids separating hepatocytes had bile accumulations and Kupffer cells. Finasteride-treated group had hepatocytes with pale staining cytoplasm, slightly large nuclei with a prominent nucleolus. Low dose LEO-treated group had hepatocytes with cytoplasmic vacuolations and densely-stained nuclei as well as wide sinusoids with moderate bile accumulation. Middle dose LEO-treated group had hepatocytes with a moderate number of pyknotic nuclei and cytoplasmic vacuolations. Sinusoids were relatively narrowed and had moderate bile accumulation. Present also in the blood vessels were karyorrhexis nuclear fragments. High dose LEO-treated group had hepatocytes with cytoplasmic vacuolations, an enlarged nucleus with a prominent nucleolus. Pyknotic nuclei and bile accumulation in the sinusoids were almost absent. Present also in the blood vessels were karyorrhexis nuclear fragments (Figure 8).

### 3.8. Effect of Treatments on Kidney Histology

**Figure 9:**
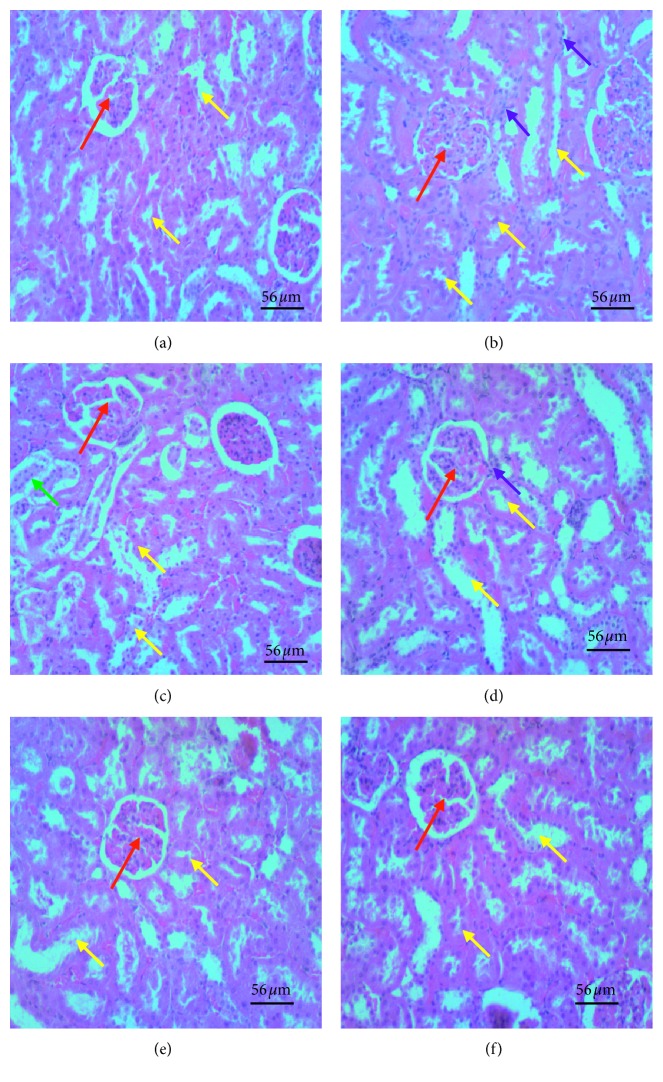
Photomicrographs of H&E stained kidney sections of various experimental groups: (a) sham; (b) model, TE (10 mg/kg)/olive oil injection (i.m.); (c) finasteride (positive control), TE (10 mg/kg)/olive oil injection (i.m.) + finasteride administration (15 mg/kg, p.o.); (d) small dose, TE (10 mg/kg)/olive oil injection (i.m.) + LEO (30 mg/kg); (e) medium dose, TE (10 mg/kg)/olive oil injection (i.m.) + LEO (100 mg/kg); (f) high dose, TE (10 mg/kg)/olive oil injection (i.m.) + LEO (300 mg/kg). The red arrow represents glomeruli with surrounding Bowman's space, the yellow arrow represents proximal and distal convoluted tubules, green represents collecting duct, and the purple arrow represents inflammatory cells. Model group showed diffuse vasodilatation and/or infiltration of inflammatory cells in the glomeruli and tubulointerstitium, enlarged glomeruli and reduced Bowman's space, tubular degenerative changes; and dilatation of medullary ray tubules. Finasteride-treated group had mild vasodilation and few enlarged glomeruli, few inflammatory cells, less tubular degenerative changes but enlarged collecting ducts. Lime essential oil-treated group showed a dose-dependent decrease in severity of these features. High dose LEO-treated group had kidney features comparable to the sham group.

Kidney micrographs of the model group showed shrunken tubules and renal corpuscles or diffuse vasodilatation and/or infiltration of inflammatory cells in the glomeruli and tubulointerstitium, tubular necrosis; and dilatation of medullary ray tubules. The finasteride-treated group had mild vasodilation and sparse enlarged renal corpuscles and glomeruli and no dilatation in medullary ray tubules. However, the glomeruli and tubulointerstitium were infiltrated with inflammatory cells, and proximal convoluted tubules had pyknotic nuclei. The lime essential oil-treated group showed a dose-dependent decrease in the severity of these features. High dose LEO-treated group had kidney features comparable to the finasteride-treated group (Figure 9).

## 4. Discussion

*Citrus aurantifolia* lime essential oil was evaluated for its ability to ameliorate variables indicative of benign prostatic hyperplasia (BPH) *in vivo*. The effect of lime essential oil on testosterone induced histological changes in selected organs including the testis, the liver, and the kidney was therefore assessed. Thus, benign prostatic hyperplasia was evaluated using serum prostate specific antigen (PSA) levels, weight, and histology of the prostate gland in this study.

Benign prostatic hyperplasia is characterized by prostatic enlargement which culminates into an increase in weight of the prostate gland [20]. In this study, testosterone-induced BPH rat groups showed a significant increase in prostate weight when compared to the sham group. A similar result of prostatic enlargement upon testosterone administration has been reported in a study by Mbaka et al. [21], on the histomorphological changes in induced benign prostatic hyperplasia with exogenous testosterone and estradiol in adult male rats. Testosterone and/or its active form dihydrotestosterone ultimately activate growth factors which regulate cell proliferation and death in cells of the prostate gland including stromal and epithelial cells of the prostate gland [22]. In BPH, TGF-*β* which promotes cell death in the prostate is downregulated while there is overexpression of KGF, EGF, and IGF which rather promotes epithelial and stromal proliferation [23–28].

When compared to the lime essential oil (LEO) and finasteride-treated groups, the model group showed a significant increase in prostate weight. Among LEO-treated group, the high dose group had significantly lesser mean prostate weight than the model group, indicative of LEO of *C. aurantifolia's p*otential of preventing or delaying prostatic hypertrophy.

Another important biomarker for the diagnosis of BPH is serum prostate specific antigen (PSA) levels. PSA is a serine protease glycoprotein which is naturally produced by epithelial cells of the prostate, and it is produced in minute amounts in the serum, but seen to be greatly elevated in BPH and prostate cancer [29]. Reduction of serum PSA levels is, therefore, indicative of the effectiveness of a trial drug or natural agent in the treatment of BPH. In this study, and prior to administration of lime essential oil and or finasteride, serum PSA levels were significantly elevated in rats which received testosterone enanthate injection, unlike the sham group which received no testosterone injection. After treatment, serum PSA levels of the model group were significantly higher than all treatment groups. This result correlates with the findings of a study done on the evaluation of *Cynanchum wilfordii* ability to ameliorate testosterone-induced benign prostatic hyperplasia [30].

The mean serum PSA levels of low and medium dose LEO treatment groups were significantly higher than that of finasteride treatment groups. On the other hand, the mean serum PSA level of the high dose LEO group was not substantially different from the finasteride-treatment group, attesting to its efficacy. One of the effects of testosterone and/or dihydrotestosterone androgen receptor complex's binding to the androgen response element is an expression of the protein PSA [31, 32]. The phytochemicals identified in LEO may actively decrease serum PSA by minimising or preventing the conversion of testosterone to dihydrotestosterone by inhibiting the enzyme 5*α*-reductase or competitively inhibiting binding of testosterone or dihydrotestosterone to the androgen receptor. Finasteride was more effective than low and medium doses of lime essential oil in terms of its ability to reduce serum PSA levels. Finasteride preferentially inhibits type II 5*α*-reductase, subsequently blocking peripheral conversion of testosterone to DHT. These resulted in low serum and tissue levels of DHT, minimal to moderate increase in serum testosterone concentrations as well as substantial increases in prostatic testosterone concentrations [33]. Finasteride is a more purified compound, unlike lime essential oil which is a cocktail of variable phytochemicals as demonstrated by the GC analyses. Thus, for a particular dose of finasteride, relatively higher doses of lime essential, as observed with the high dose, will be needed to exert a comparable effect. This could account for why low doses of the lime essential oil could not produce results similar to finasteride. This notwithstanding lime essential oil is readily available and relatively cheaper compared finasteride.

Additionally, restoration of prostate histoarchitecture is an important parameter used in various studies to assess the potency of anti-BPH agents. So far, no known study has commented on the association between prostatic secretion and BPH development. In this study, BPH-untreated group produced more prostatic secretions, compared to all BPH-treatment and the sham groups. More so, glandular epithelial thickening of the glands irrespective of the lobes was more pronounced in the model group than lime essential oil- and finasteride-treated groups. Androgens like testosterone and dihydrotestosterone (DHT) are known to be associated with the regulation of cell proliferation, specifically, in androgen-dependent organs like the prostate and seminal vesicles [31]. Exogenous testosterone, administered is often converted to DHT, and after binding to androgen receptors increase transcription of androgen-dependent genes to stimulate protein synthesis. The overall mechanism leads to the proliferation of cells, by stimulating transcription of growth factors which are mitogenic for the epithelial and stromal cells of the prostate, hence the thickening of the glandular epithelium [17].

BPH is known to increase the presence of free radicals in serum; therefore, the ability of the investigated plant extract to reduce these free radicals is essential for the treatment of BPH. Hypotestosteronemia is reported to cause a buildup of oxidative radical species in tissues [32] by impairing the activity of three main antioxidant enzymes, namely, catalase, superoxide dismutase, and glutathione peroxidase in human testes [33], or downregulate the activity of prooxidant enzymes (i.e. NADPH-oxidase) in the prostate [34, 35]. In this study, the total antioxidant capacity (TAC) of serum was used to quantify residual antioxidants in the serum. The model group had the least serum TAC while lime essential oil-treated groups recorded a dose-dependent increase in serum TAC levels. Antioxidant potential of lime essential oil has been confirmed *in vitro* using egg albumin assay and red blood cell model, and thus, ingestion of the oil could add to the plasma antioxidants thereby causing the lime essential oil-treated groups to have higher plasma TAC. The buildup of oxygen radicals in tissue or plasma results in damage to the histology of a tissue [36, 37].

Testosterone has an effect on several body organs including the kidney [16, 38–41], testis, and liver [39, 40]. Therefore the impact of hypotestosteronemia on histology of selected organs of the body was assessed. The model group recorded more histological damages than oil- and finasteride-treated groups. Histological changes observed in the study could be attributable to testosterone engendered oxidative stress-mediated damage. Serum testosterone was not determined, but the serum PSA level has a positive correlation with serum testosterone level. The finasteride-treated group had lesser serum PSA, and thus, this group is likely to record low serum testosterone thereby minimizing testosterone-mediated histological changes. The oil- treated group also recorded a dose dependent decrease in histological changes with the high dose oil-treated group having better histological outcomes than lower and mid-dose groups.

## 5. Conclusion

Lime essential oil from unripe *Citrus aurantifolia* has demonstrated potent anti-benign prostatic hyperplastic activity by causing significant reduction in serum PSA levels, minimizing prostate gland enlargement and also reverted testosterone-induced histological changes in experimental rats. Lime essential oil from *Citrus aurantifolia* could potentially be used as an alternative drug to manage benign prostatic hyperplasia, and therefore should be investigated further to derive its full potential as a drug. Thus, the individual phytochemicals need to be investigated to establish the particular phytochemical exerting particular bioactivity and the mechanism of action and whether there is a synergy among them.

## Figures and Tables

**Table 1:** Percentage composition and retention time of chemical composition of unripe lime essential oil.

Peak no.	Components	Formulae	Measured (*m*/*z*) [M + H]^+^	Relative (%)	Retention time (min.)	Main product ions [M + H]^+^
1	Pinene	C_10_H_16_	136	14.3	5.05	136, 121, 107, 93, 81, 79, 69, 43
2	Germacrene A	C_15_H_26_	207	49.0	6.08	206, 136, 121, 106, 93, 68, 44
3	Germacrene B	C_15_H_26_	207	12.2	6.79	206, 136, 121, 106, 93, 68, 44
4	Linalool dimer	2(C_10_H_18_O)	309	2.9	7.52	110, 94, 79, 59, 44, 40
5	Anisole	C_7_H_8_O	110	1.1	9.64	110, 94, 84, 79, 77, 69, 59
6	Bornane	C_10_H_18_	139	11.3	10.32	139, 136, 121, 107, 93, 81, 43
7	Anethole	C_10_H_12_O	148	1.5	11.67	147, 119, 108, 94, 69, 55
8	Citral/geranial	C_10_H_16_O	152	2.9	12.48	152, 137, 123, 109, 94, 69
9	Safrole	C_10_H_10_O_2_	161	0.3	14.48	161, 136, 121, 108, 93, 77, 55
10	Demitol	C_9_H_20_O	145	0.6	15.04	145, 128, 114, 97, 83, 69, 57, 44

## Data Availability

The data used to support the findings of this study are available from the corresponding author upon request through dacheampong@ucc.edu.gh.

## Supplementary Materials

Supplementary Materials Chemical composition of the unripe lime essential oil GC/MS analysis identified about ten (10) phytochemicals as its major constituents. The single ion monitoring (SIM) chromatograms generated via accurate *m*/*z* representation of terpenes in literature [1] were employed to identify respective MS spectra of essential oil components and their molecular formulae were also computed on the basis that the isotopic fit ratios (iFit) were assumed to be close to zero as possible (Figures S1–S7). Schemes S1–S7 and Figures S1–S7 depicts fragmentation mechanistic pattern and GC/MS spectra of individual components of the oil detailed explained in the main text. In summary, two isomers of Germacrene isomers were the major components (61.2%) followed by Pineen (14%). The remaining chemical compounds were in trace amounts as listed in Table 1 in the main text. The peaks labeled as **2** and **3** in the GC spectrum with *m*/*z* 207 are isomers of germacrene with a typical fragmentation pattern depicted in the MS spectrum (Figure S2 and Scheme S2) comparable to literature data [1, 2]. The fragmentation mechanisms of all the compounds were proposed to further confirm their presence (Scheme S2). For germacrene isomers, the first step in fragmentation reactions seems to be breaking a single bond through the loss of an electron from the molecule **2**. Molecule **2** with retention time (Rt) of 6.10 min. (Figure S2) produced a precursor ion at *m*/*z* 207 [M + H] (C_15_H_26_) due to completely breakdown of a *π*-bond by loss of an electron. This led to loss of an isopropyl group. The fragmentation of this molecule (Figure S2, Scheme S2) gave molecular ion at *m*/*z* 136, derived from the loss of two methyl (−30 Da) groups and isopropyl group (seventy-one mass units (−71 Da) after a possible 1,2 methyl rearrangement of dimethyl derivative of germacrene to a more stable derivative of cyclooctene-dimer (Scheme S2). Products ions at *m*/*z* 93 corresponds to the ring opening of the cyclooctene-dimer and the loss of an *m*/*z* 44 neutral fragment to form the base peak with 100%. Similarly, fragmentation mechanism of all other components (Pineen, Anisole, Anethole, Safrole and Demitol) were proposed to further confirm their presence (Scheme S1, S4–S7). Table S1: effect of LEO on prostate weight.
