# Supplementary material for: Histoprotective Effect of Essential Oil from Citrus aurantifolia in Testosterone-Induced Benign Prostatic Hyperplasia Rat
Source: Adv Urol. 2019 Sep 25;2019:3031609. doi: 10.1155/2019/3031609 (PMC6778952; doi:10.1155/2019/3031609)
Supplement: Supplementary Materials — Chemical composition of the unripe lime essential oil GC/MS analysis identified about ten (10) phytochemicals as its major constituents. The single ion monitoring (SIM) chromatograms generated via accurate m/z representation of terpenes in literature [1] were employed to identify respective MS spectra of essential oil components and their molecular formulae were also computed on the basis that the isotopic fit ratios (iFit) were assumed to be close to zero as possible (Figures S1–S7). Schemes S1–S7 and Figures S1–S7 depicts fragmentation mechanistic pattern and GC/MS spectra of individual components of the oil detailed explained in the main text. In summary, two isomers of Germacrene isomers were the major components (61.2%) followed by Pineen (14%). The remaining chemical compounds were in trace amounts as listed in Table 1 in the main text. The peaks labeled as 2 and 3 in the GC spectrum with m/z 207 are isomers of germacrene with a typical fragmentation pattern depicted in the MS spectrum (Figure S2 and Scheme S2) comparable to literature data [1, 2]. The fragmentation mechanisms of all the compounds were proposed to further confirm their presence (Scheme S2). For germacrene isomers, the first step in fragmentation reactions seems to be breaking a single bond through the loss of an electron from the molecule 2. Molecule 2 with retention time (Rt) of 6.10 min. (Figure S2) produced a precursor ion at m/z 207 [M + H] (C15H26) due to completely breakdown of a π-bond by loss of an electron. This led to loss of an isopropyl group. The fragmentation of this molecule (Figure S2, Scheme S2) gave molecular ion at m/z 136, derived from the loss of two methyl (−30 Da) groups and isopropyl group (seventy-one mass units (−71 Da) after a possible 1,2 methyl rearrangement of dimethyl derivative of germacrene to a more stable derivative of cyclooctene-dimer (Scheme S2). Products ions at m/z 93 corresponds to the ring opening of the cyclooctene-dimer and the loss of an m/z 44 neut [file 3031609.f1.docx]

**Supplementary documents**

**Histoprotective effect of essential oil from *Citrus aurantifolia* in testosterone-induced benign prostatic hyperplasia rat**

**Running Title: Host protective effect of essential oil from Citrus aurantifolia**

# Desmond O Acheampong^1*,^ Isaac K Barffour^1^, Alex Boye^2^, Ernest A Asiamah^1^, Francis A Armah^1^, Christian K. Adokoh^1^, Joy F Oluyemi^1^, Benjamin Adrah^1^, Richard Opoku^1^, and Emmanuel Adakudugu^1^

^1^Department of Biomedical Sciences, School of Allied Health Sciences, University of Cape Coast, Ghana.

^2^Department of Medical Laboratory Technology, School of Allied Health Sciences, University of Cape Coast, Ghana.

***Corresponding author:** Desmond O Acheampong, Department of Biomedical Sciences, School of Allied Health Sciences, University of Cape Coast, Cape Coast, Ghana; Tel: +233 543710234; Email: dacheampong@ucc.edu.gh. do.acheampong@gmail.com.

**Chemical Composition of the Unripe Lime Essential oil**

GC/MS analysis of unripe lime essential oil identified about ten (10) phytochemicals as its major constituents. The single ion monitoring (SIM) chromatograms generated via accurate m/z representation of terpenes in literature [1] was employed to identify respective MS spectra of essential oil components and their molecular formulae were also computed on the basis that the isotopic fit ratios (iFit) was assumed to be close to zero as possible (Figure S1-7). The schemes and figures below (Scheme 1-7 and Fig. S1-7) depicts fragmentation mechanistic pattern and GC/MS spectra of individual components of the oil detailed explained in the main text. In summary, two isomers of Germacrene isomers were the major components (61.2%) followed by Pineen (14%).The remaining chemical compounds were in trace amounts as listed in table 1 in the main text. The peaks labeled as **2** and **3** in the GC spectrum with m/z 207 are isomers of germacrene with a typical fragmentation pattern depicted in the MS spectrum (Figure 2 & Scheme 2) comparable to literature data [1, 2]. The fragmentation mechanisms of all the compounds were proposed to further confirm their presence (Scheme 2). For germacrene isomers, the first step in fragmentation reactions seems to be breaking a single bond through the loss of an electron from the molecule **2**. Molecule **2** with retention time (Rt) of 6.10 min (Fig. 2) produced a precursor ion at m/z 207 [M+H] (C_15_H_26_) due to completely breakdown of a π-bond by loss of an electron. This led to loss of an isopropyl group. The fragmentation of this molecule (Figure 2, Scheme 2) gave molecular ion at m/z 136, derived from the loss of two methyl (-30 Da) groups and isopropyl group (seventy one mass units (-71 Da) after a possible 1,2 methyl rearrangement of dimethyl derivative of germacrene to a more stable derivative of cyclooctene-dimer (Scheme 2). Products ions at m/z 93 corresponds to the ring opening of the cyclooctene-dimer and the loss of an m/z 44 neutral fragment to form the base peak with 100%. Similarly, fragmentation mechanism of all other components (Pineen,Anisole,Anethole, Safrole and Demitol) were proposed to further confirm their presence (Scheme 1, 4-7).


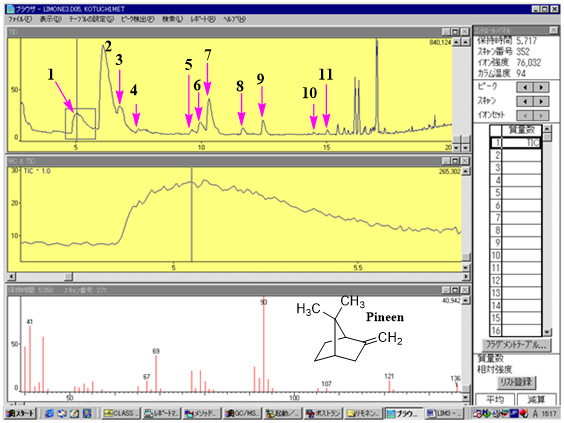


Figure S1: Typical GC and mass spectrum of the fragmentation pattern of Pineen

Scheme S1: Proposed fragmentation pathways of Pineen


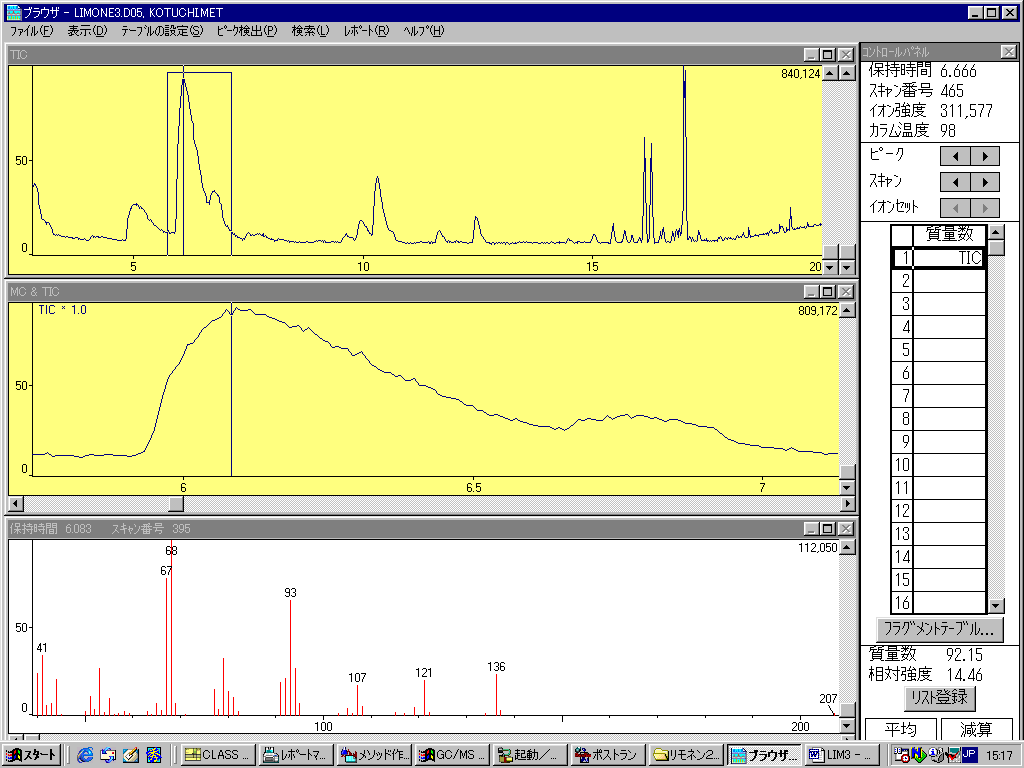


Figure S2: Typical GC and mass spectrum of the fragmentation pattern of germacrene A

Scheme S2: Proposed fragmentation pathways of germacrene A


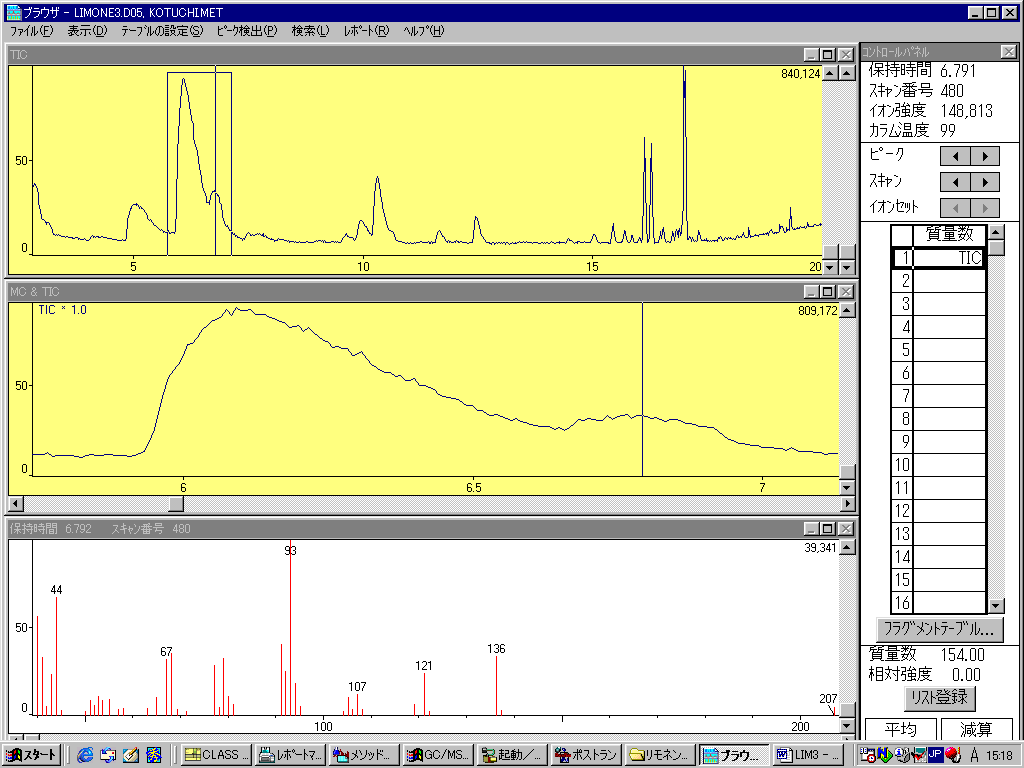


Figure S3: Typical GC and mass spectrum of the fragmentation pattern of germacrene B

Scheme S3: Proposed fragmentation pathways of germacrene B


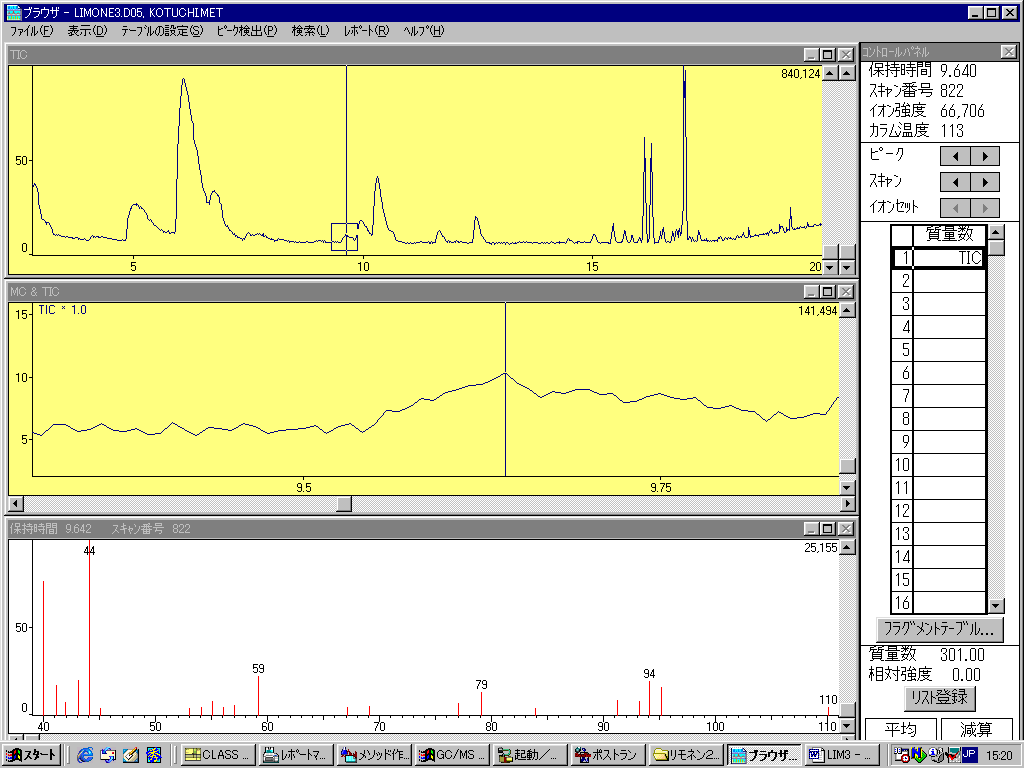

Figure S4: Typical GC and mass spectrum of Anisole and the fragmentation pattern

Scheme S4: Proposed fragmentation pathways of Anisole


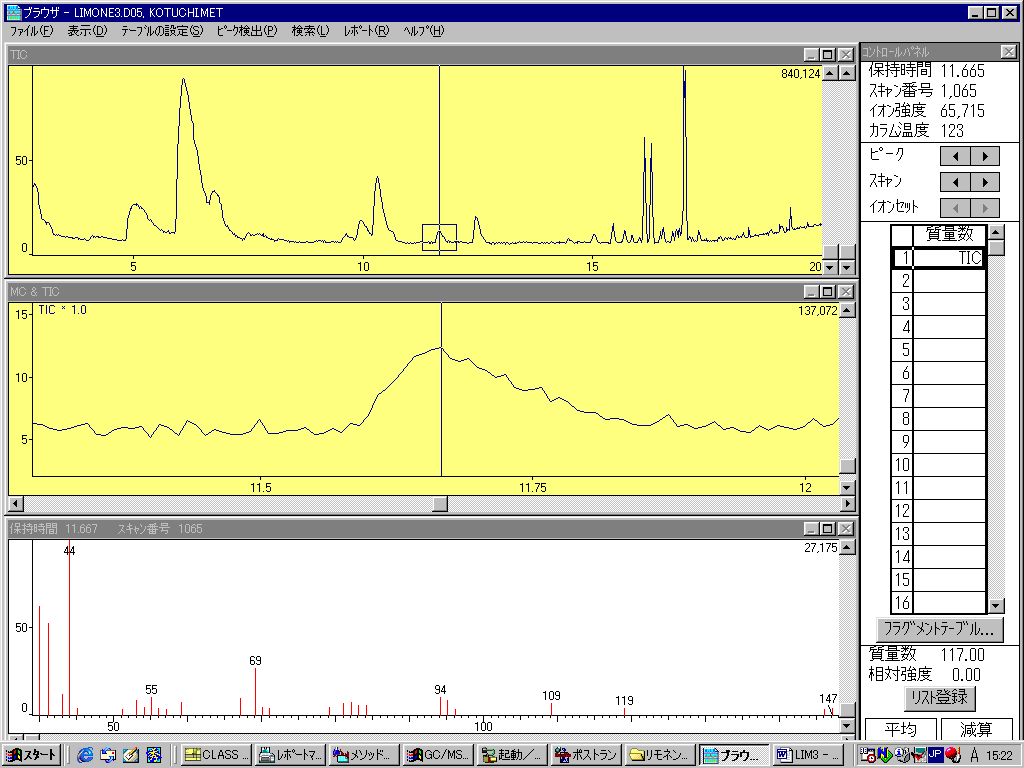

Figure S5. Typical GC and mass spectrum of Anethole and the fragmentation pattern

Scheme S5: Proposed fragmentation pathways of Anethole


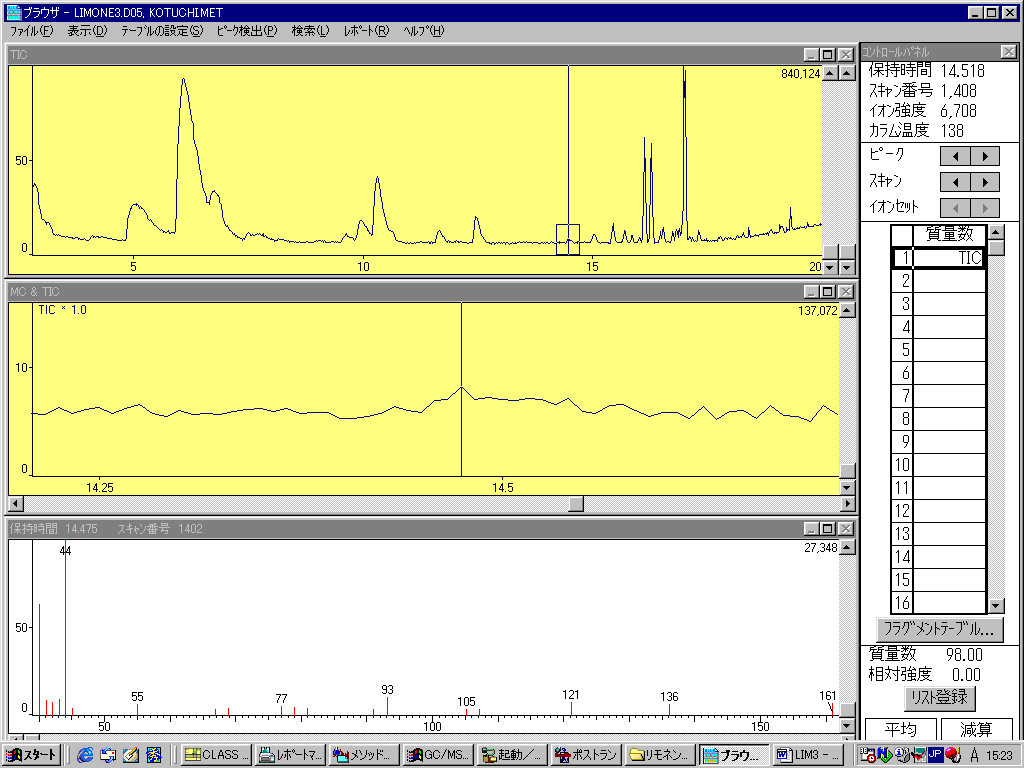

Figure S6: Fragmentation pattern of Safrole with GC and mass spectrum

Scheme S6. Fragmentation pattern of Safrole typical of reported or theoretical values [2].


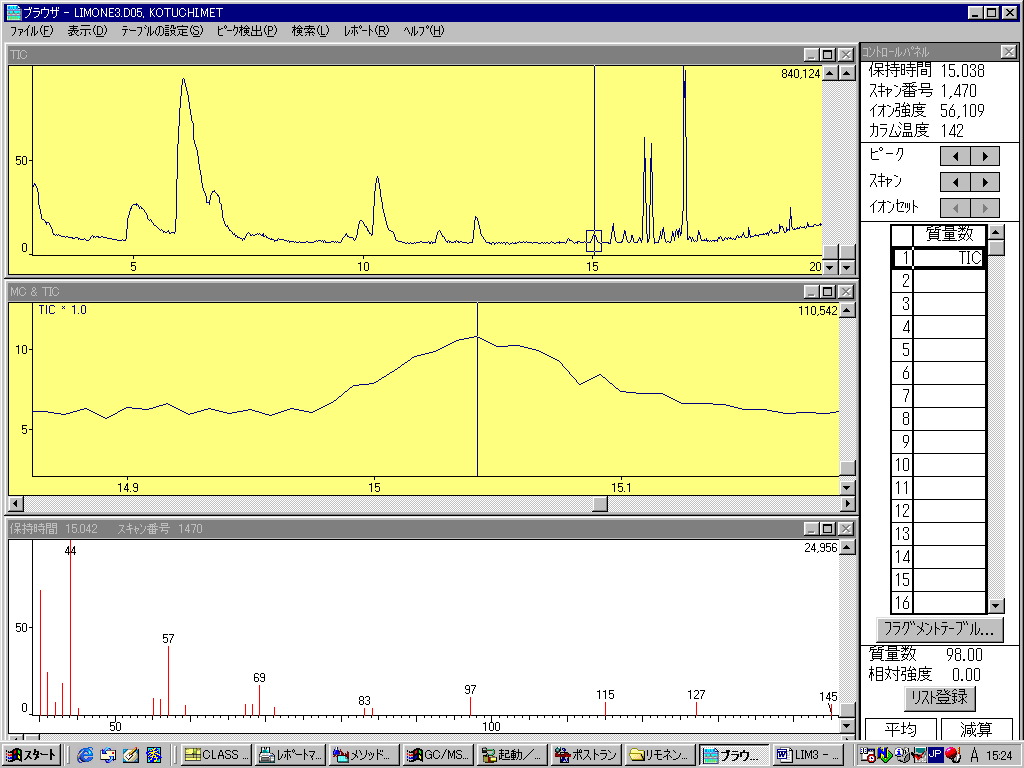

Figure S7. Fragmentation pattern of Demitol with GC and mass spectrum

Scheme S7. Fragmentation pattern of Demitol

# Table S1. Effect of LEO on prostate weight

| Group | Mean prostate weight ± error (g) | p-value | Comparison with sham (C) |
| --- | --- | --- | --- |
| Sham (C) | 0.333 ± 0.018 |  | C:NC-155.93% (p=0.0001)* |
| Model (NC)  LD  MD  HD | 0.853 ± 0.091  0.657 ± 0.023  0.785 ± 0.026  0.600 ± 0.003 |  | C:LD - 97.12% (p=0.0246)*  C:MD-135.52% (p=0.0008)*  C:HD - 80.02% (p=0.0803)  C:PC - 6.66% (p=0.9998) |
| Finasteride (PC) | 0.356 ± 0.008 |  |  |

LD – low dose group; MD – medium dose; HD – high dose * denotes statistical significance (p˂0.05)

References

[1] Sydow von. E.(1964) Mass Spectrometry of Terpenes, Acta chem. Scand. 18, no.5.

[2]National institute of standard technology (NIST)(2016).. Material measurement laboratory, NIST Chemistry WebBook. NIST MS number 229569. <http://webbook.nist.gov/cgi/cbook.cgi?ID=C94597&Mask=200>
